# Unraveling the Genetic Links: The Impacts of Antidiabetic Drugs on Stroke Risk

**DOI:** 10.31083/RCM40051

**Published:** 2025-10-23

**Authors:** Jie Chen, Yu-Zhen Wang, Liu-Cheng Li, Jiadong Xu

**Affiliations:** ^1^Department of Pharmacy, Sir Run Run Shaw Hospital, School of Medicine, Zhejiang University, 310016 Hangzhou, Zhejiang, China; ^2^Department of Physiology, Zhejiang Chinese Medical University, 310053 Hangzhou, Zhejiang, China

**Keywords:** antidiabetic drugs, stroke, Mendelian randomization, SGLT2 inhibitors, genetic epidemiology

## Abstract

**Background:**

Diabetes is widely recognized as a major contributor to stroke risk. Certain antidiabetic medications have demonstrated promising effects on reducing stroke incidence and improving outcomes.

**Methods:**

To evaluate the potential impacts of antidiabetic drugs comprehensively, we developed an analytical framework that incorporates summary-based Mendelian randomization (SMR), two-sample Mendelian randomization (TSMR), and colocalization analyses. Summary statistics from the largest genome-wide association study (GWAS) of stroke and known subtypes were utilized, along with gene expression data from the blood, aorta, coronary artery, and tibial artery provided by the eQTLGen or GTEx V8 consortia.

**Results:**

Elevated expression levels of the solute carrier family 5 member 2 (*SLC5A2*) gene, which is targeted by sodium–glucose co-transporter 2 (SGLT2) inhibitors, in the tibial artery and the potassium inwardly rectifying channel subfamily J member 11 (*KCNJ11*) gene, which is targeted by sulfonylureas, in the blood were linked to an increased risk of any ischemic stroke (AIS) (*KCNJ11*: odds ratio (OR) = 1.11, 95% confidence interval (CI) = 1.01–1.21; *p* = 0.033; *SLC5A2*: OR = 1.05, 95% CI = 1.01–1.10; *p* = 0.017, respectively), according to the SMR analysis. Additionally, the upregulation of insulin receptor (*INSR*) expression in the tibial artery was associated with a reduction in stroke incidence in patients with large-artery atherosclerotic stroke (LAS) (OR = 0.66, 95% CI = 0.46–0.95; *p* = 0.026). The TSMR results were consistent with these findings. Furthermore, the expressions of *SLC5A2* and *INSR*, which are associated with AIS and LAS, respectively, were colocalized in the tibial artery.

**Conclusion:**

Our findings suggest that SGLT2 inhibitors and sulfonylureas may influence the risk of AIS. Additionally, activation of the insulin receptor may reduce the risk of LAS. These results increase our understanding of medication options for stroke patients who require hypoglycemic agents and provide a basis for the strategic repurposing of antidiabetic drugs.

## 1. Introduction

Stroke remains a predominant cause of mortality and long-term disability worldwide, thus presenting a significant public health challenge [1]. This challenge is especially pronounced in individuals with metabolic conditions such as diabetes mellitus, which not only increases susceptibility to heart-related illnesses but also makes managing health outcomes more complex [2]. Therefore, understanding the relationships between diabetes management, especially through the use of antidiabetic medications, and stroke risk is essential for developing effective preventive and therapeutic strategies.

Type 2 diabetes (T2D) is a known risk factor for stroke [3]. For example, a study reported that the incidence of stroke among individuals with diabetes could reach 34.4%, with ischemic stroke accounting for 85.2% of cases [4]. Multiple studies have also demonstrated a strong correlation between high blood glucose levels and elevated stroke risk [5, 6]. Thus, maintaining stable blood glucose levels is vital for patients with stroke, as diabetes not only increases stroke susceptibility but also worsens clinical outcomes, increasing both mortality and severe complications [7]. Persistent hyperglycemia enhances atherogenesis and facilitates thrombosis by triggering endothelial dysfunction, oxidative stress, and inflammation, all of which jointly increase the risk of ischemic stroke in patients with diabetes [8]. Antidiabetic drugs, through various mechanisms and metabolic effects, may influence vascular risk factors and offer potential stroke protection.

However, the link between antidiabetic drugs and stroke risk is intricate and varies by drug class. Indeed, while insulin effectively controls hyperglycemia, it can also increase the risk of cardiovascular events by causing hypoglycemia. In contrast, modern therapies such as glucagon-like peptide-1 (GLP-1) receptor agonists and sodium–glucose co-transporter 2 (SGLT2) inhibitors aid in regulating blood sugar and offer additional cardiovascular benefits [9, 10, 11], including reduced blood pressure, ameliorated inflammation, and improved lipid profiles. These distinct drug effects necessitate a detailed exploration of their influence on stroke risk. Recent advances in genetic epidemiology, particularly Mendelian randomization (MR) [12], have provided robust tools to elucidate these relationships. MR utilizes genetic variants as proxies for drug exposure, thereby minimizing confounding factors inherent in observational studies. Integrating these genetic insights with data from randomized controlled trials and large-scale cohort studies offers a powerful framework for assessing the true impacts of antidiabetic medications on stroke risk.

This study aimed to employ summary-based Mendelian randomization (SMR) [13], two-sample Mendelian randomization (TSMR; https://mrcieu.github.io/TwoSampleMR/index.html), and colocalization analyses [14] to explore the causal links between antidiabetic drug targets and stroke subtypes. Our goal was to enhance the understanding and clinical application of antidiabetic medications to reduce cardiovascular risk in individuals with diabetes.

## 2. Methods

This study complied with the Strengthening the Reporting of Observational Studies in Epidemiology Using Mendelian Randomization (STROBE-MR) guidelines. For further details, please refer to the STROBE-MR Checklist Table provided in the **Supplementary Materials** [15].

### 2.1 Study Design

**Fig. 1.**
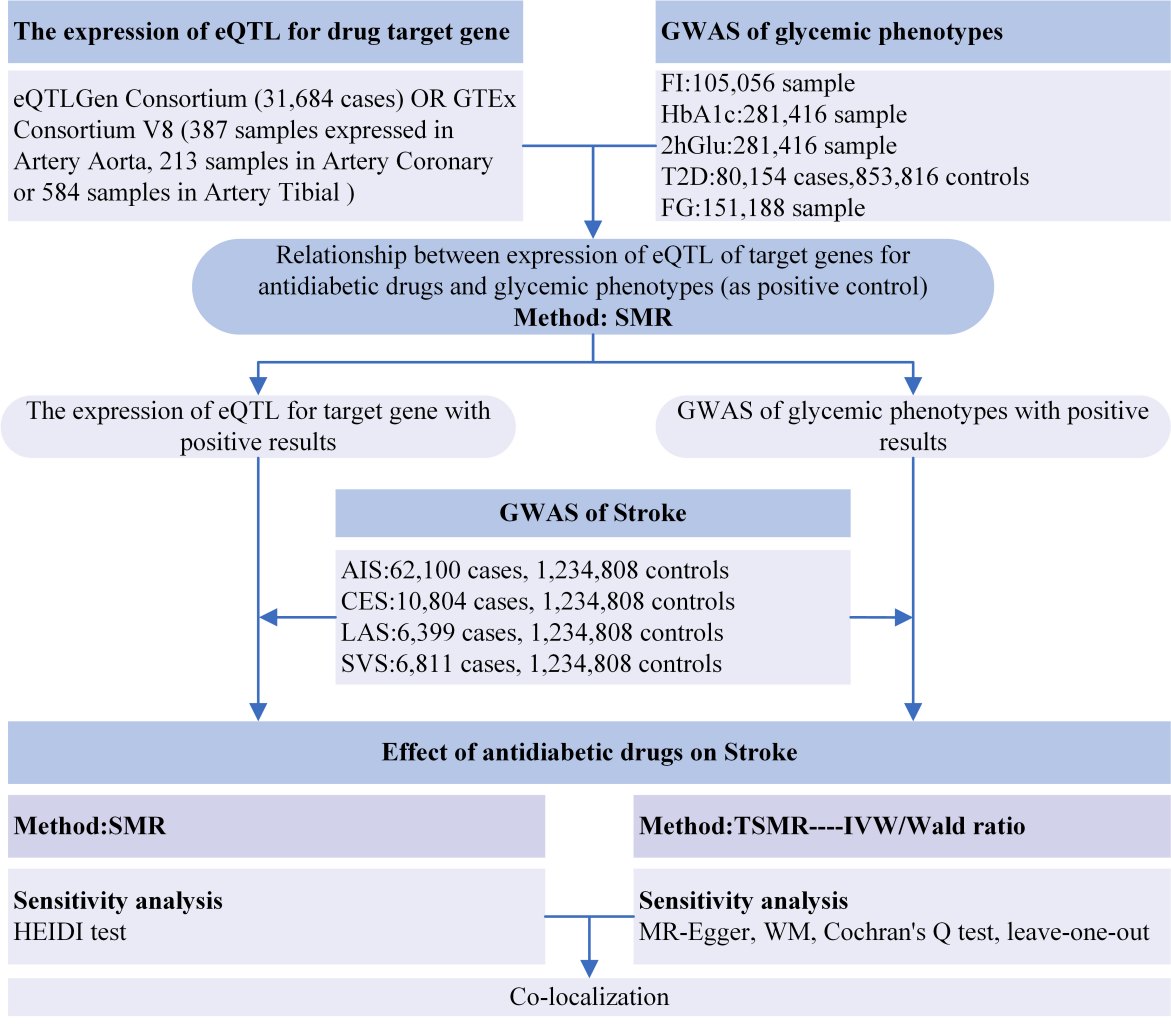
**Flowchart of the study design**. Abbreviations: eQTLs, expression quantitative trait loci; GWAS, genome-wide association study; FI, fasting insulin; HbA1c, glycated hemoglobin; 2hGlu, 2-hour glucose postchallenge; T2D, type 2 diabetes; FG, fasting glucose; AIS, any ischemic stroke; CES, cardioembolic stroke; LAS, large-artery atherosclerotic stroke; SVS, small-vessel stroke; SMR, summary-based Mendelian randomization; TSMR, two-sample Mendelian randomization; HEIDI, heterogeneity in dependent instruments; IVW, inverse-variance weighted; WM, weighted median.

The SMR and TSMR methodologies were applied to assess the influence of antidiabetic medications on stroke and related subtypes. This analysis followed three key assumptions: (1) genetic variants should exhibit a strong correlation with the exposure; (2) these variants must remain independent of confounding factors that might distort the exposure‒outcome relationship; (3) any effects on the outcome must be mediated solely through exposure, without direct influence. Colocalization analyses were subsequently conducted to strengthen evidence for significant associations. Fig. 1 presents the flowchart of the study design.

### 2.2 Effects of Antidiabetic Drug Targets on Stroke

#### 2.2.1 Data Sources

The primary antidiabetic pharmacological agents included metformin, GLP-1 receptor agonists, SGLT2 inhibitors, dipeptidyl peptidase-4 (DPP-4) inhibitors, insulin and its analogs, thiazolidinediones, and sulfonylureas [16]. The target genes of these drugs were identified using the DrugBank [17] and ChEMBL [18] databases. Due to the complex and variable mechanisms of action of metformin across these databases, metformin was excluded from further analysis (details provided in Table 1).

**Table 1.** **Target genes of antidiabetic drugs from DrugBank**.

Drug class	DrugBank encoding genes	Gene location
Metformin	*PRKAB1*	NA
	*ETFDH*	NA
GLP-1 receptor agonists	*GLP1R*	Chr6: 39,016,557–39,059,079
SGLT2 inhibitors	*SLC5A2*	Chr16: 31,494,323–31,502,181
DPP-4 inhibitors	*DPP-4*	Chr2: 162,848,755–162,930,904
Insulin and its analogs	*INSR*	Chr19: 7,112,266–7,294,425
Thiazolidinediones	*PPARG*	Chr3: 12,328,867–12,475,855
Sulfonylureas	*KCNJ11*	Chr11: 17,386,719–17,410,878
	*ABCC8*	Chr11: 17,414,045–17,498,441

The gene locations in the human assembly GRCh37 were obtained from https://grch37.ensembl.org. GLP-1, glucagon-like peptide-1; SGLT2, sodium‒glucose co-transporter 2; DPP-4, dipeptidyl peptidase-4; NA, not applicable; INSR, insulin receptor; PRKAB1, protein kinase AMP-activated non-catalytic subunit beta 1; ETFDH, electron transfer flavoprotein dehydrogenase; PPARG, peroxisome proliferator activated receptor gamma; ABCC8, ATP binding cassette subfamily C member 8; SLC5A2, solute carrier family 5 member 2; KCNJ11, potassium inwardly rectifying channel subfamily J member 11.

Gene expression quantitative trait loci (eQTLs) linked to the target genes of drugs were retrieved from the eQTLGen consortium (https://www.eqtlgen.org/) dataset, which included data from blood samples of 31,684 individuals. The cis-eQTLs were extracted from this dataset to characterize the effects of these drugs. Tissue-specific gene expression and stroke susceptibility were analyzed using GTEx (V8) cis-eQTL summary statistics, which are accessible at https://yanglab.westlake.edu.cn/data/SMR/GTEx_V8_cis_eqtl_summary.html. For this analysis, we focused on samples from the aorta, coronary artery, and tibial artery in the GTEx dataset. Details of the eQTL data are provided in **Supplementary Table 1**.

Antidiabetic agents primarily manage T2D by reducing blood glucose and glycated hemoglobin (HbA1c) levels. Therefore, genome-wide association study (GWAS) data on fasting insulin (FI) (105,056 cases) [19], fasting glucose (FG) (151,188 cases) [19], HbA1c (281,416 cases) [20], 2-hour glucose postchallenge (2hGlu) (281,416 cases) [20], and T2D (80,154 cases/853,816 controls) [21] were used as positive controls. Genes with significant associations were included in the SMR analysis. For the stroke data, we used a fixed-effects inverse-variance weighted (IVW) GWAS meta-analysis [22], which included 62,100 cases of any ischemic stroke (AIS) and 1,234,808 controls. Subtypes of ischemic stroke, including cardioembolic stroke (CES: 10,804 cases), large-artery atherosclerotic stroke (LAS: 6399 cases), and small-vessel stroke (SVS: 6811 cases), are shown in **Supplementary Table 2**. These subtypes constitute the majority of global ischemic strokes [23]. The LAS subtype corresponds to large-vessel atherosclerosis, SVS denotes cerebral small-vessel disease, and CES signifies cardiac embolism sources. This categorization is consistent with the established Trial of Org 10172 in Acute Stroke Treatment (TOAST) classification criteria [24].

The GWAS summary data for FI, FG, HbA1c, and 2hGlu, which revealed statistically significant SMR results with cis-eQTLs of target genes, were used as proxies. In the analysis of selected cis-eQTL genes, single-nucleotide polymorphisms (SNPs) with *p*-values < 5 × 10⁻⁸ and weak linkage disequilibrium (r^2^ <0.1) within a ± 100 megabase (Mb) region around the target gene were selected as instrumental variables (IVs).

#### 2.2.2 Statistical Analysis

SMR software (version 1.02, https://yanglab.westlake.edu.cn/software/smr/#Overview) [13] was used to harmonize and analyze the data. For TSMR, cis-eQTLs served as IVs, with the IVW method used as the primary analytical approach. The Wald ratio method was applied when a single IV was available.

#### 2.2.3 Sensitivity Analysis

SMR version 1.02 was used for the TSMR analysis, which incorporated SNPs that met the significance threshold (*p* < 0.05). The heterogeneity in dependent instruments (HEIDI) test, with a *p*-value threshold of 0.01, was conducted to differentiate pleiotropy from linkage. For TSMR, sensitivity analyses included the MR‒Egger and weighted median (WM) methods, which were contingent upon possessing a sufficient number of IVs. Heterogeneity was assessed using Cochran’s Q test, and the leave-one-out approach was employed to evaluate the impact of individual SNPs.

Colocalization analysis was used to determine whether stroke-related SNPs and gene expression data were associated with a common causal genetic variant. A Bayesian framework was applied in this analysis to compute the posterior probabilities for five hypotheses (PPHs): H0 (no link to either trait), H1 (exclusive association with the first trait), H2 (exclusive association with the second trait), H3 (separate causal variants for each trait), and H4 (a common causal variant shared by both characteristics). A PPH4 value >0.6 indicated colocalization between gene expression targets and stroke.

## 3. Results

### 3.1 Genetic Instruments

This study considered principal antidiabetic pharmacotherapies, including metformin, GLP-1 receptor agonists, SGLT2 inhibitors, DPP-4 inhibitors, insulin and its analogs, thiazolidinediones, and sulfonylureas. Genes encoding the target proteins for these drugs were identified via the DrugBank and ChEMBL databases (Table 1). Nine genes were associated with HbA1c (**Supplementary Table 3**), T2D (**Supplementary Table 4**), fasting blood glucose (**Supplementary Table 5**), or 2hGlu (**Supplementary Table 6**), whereas no genes were associated with FI (**Supplementary Table 7**).

### 3.2 Causal Associations Between Stroke and Antidiabetic Drug Targets

**Fig. 2.**
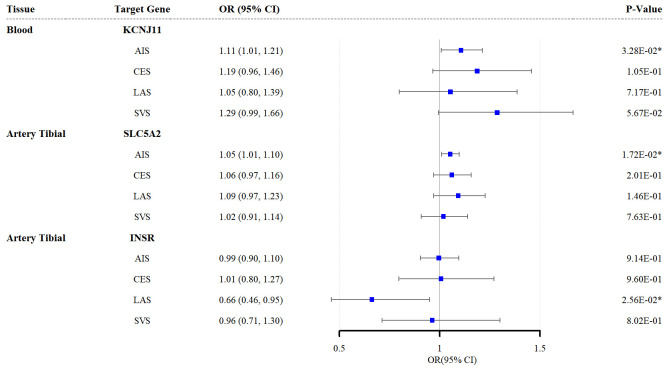
**Forest plots of the effects of the three antidiabetic drug targets on the risk of the four stroke subtypes (results based on the SMR analysis)**. * indicates *p* < 0.05. SMR, summary-based Mendelian randomization; AIS, any ischemic stroke; CES, cardioembolic stroke; LAS, large-artery atherosclerotic stroke; SVS, small-vessel stroke; SLC5A2, solute carrier family 5 member 2; KCNJ11, potassium inwardly rectifying channel subfamily J member 11; INSR, insulin receptor.

According to the SMR analysis, genetic variations in the target genes for sulfonylureas (*KCNJ11*) and SGLT2 inhibitors (*SLC5A2*) were significantly associated with AIS risk (*KCNJ11*: odds ratio (OR) = 1.11, 95% confidence interval (CI) = 1.01–1.21; *p* = 0.033; *SLC5A2*: OR = 1.05, 95% CI = 1.01–1.10; *p* = 0.017). Furthermore, the upregulation of insulin receptor (*INSR*) expression in the tibial artery was associated with LAS mitigation (OR = 0.66, 95% CI = 0.46–0.95; *p* = 0.026) (Fig. 2). The results for other target genes and their associations with stroke subtypes are detailed in **Supplementary Tables 8–11**. Additionally, the SMR analysis with HEIDI tests revealed no evidence of pleiotropy for the three target genes of the respective antidiabetic drugs in relation to stroke.

**Fig. 3.**
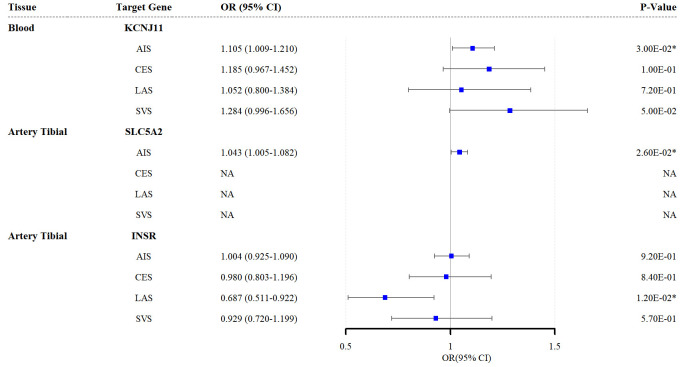
**Forest plots of the effects of the three antidiabetic drug targets on the risk of the four stroke subtypes (results based on the TSMR analysis)**. * indicates *p* < 0.05. TSMR, two-sample Mendelian randomization; AIS, any ischemic stroke; CES, cardioembolic stroke; LAS, large-artery atherosclerotic stroke; SVS, small-vessel stroke; SLC5A2, solute carrier family 5 member 2; KCNJ11, potassium inwardly rectifying channel subfamily J member 11; INSR, insulin receptor.

The TSMR analysis findings corroborated those of the SMR analysis, showing that elevated *KCNJ11* expression in the blood and *SLC5A2* expression in the tibial artery increased AIS risk (*KCNJ11*: OR_ivw_ = 1.105, 95% CI = 1.009–1.210; *p* = 0.030; *SLC5A2*: OR_ivw_ = 1.043, 95% CI = 1.005–1.082; *p* = 0.026). Similarly, upregulated *INSR* expression in the tibial artery reduced LAS risk (OR_ivw_ = 0.687, 95% CI = 0.511–0.922; *p* = 0.012; Fig. 3). Additional TSMR results are provided in **Supplementary Tables 12–15**. No SNP agent was inadequate for heterogeneity tests, and the leave-one-out analysis results are presented in **Supplementary Tables 16–19**.

### 3.3 Colocalization Analysis

Next, colocalization analysis was conducted to assess whether SNPs linked to stroke and eQTLs share causal genetic variants. These results suggested that in the region, most identified genes likely possessed a common causal variant. Notably, significant colocalization was observed for *INSR* and *SLC5A2* across datasets (PPH4 = 0.77 and 0.60, respectively), whereas *KCNJ11* did not reach statistical significance (**Supplementary Table 20**).

## 4. Discussion

This study employed the SMR and TSMR methodologies to clarify the influence of antidiabetic drug targets on stroke risk. The significant links observed between specific drug targets and the risk of stroke suggest that certain antidiabetic medications might provide therapeutic benefits for stroke prevention or management. Specifically, increased expression of *KCNJ11* (the target gene of sulfonylureas) and *SLC5A2* (the target gene of SGLT2 inhibitors) was associated with a heightened risk of ischemic stroke. In contrast, upregulated *INSR* expression was linked to a reduced risk of LAS.

### 4.1 KCNJ11 and Sulfonylureas

The *KCNJ11* gene encodes the Kir6.2 subunit, a key component in the ATP-sensitive potassium (K-ATP) channel. Sulfonylureas inhibit the K-ATP channel by binding to its SUR1 subunit, thereby promoting insulin secretion from pancreatic beta cells. Despite the longstanding use of sulfonylureas in the management of T2D, which is due to the efficacious glucose-lowering properties of these compounds, the relationship between these compounds and stroke remains controversial. A previous study demonstrated that sulfonylureas did not affect the severity of stroke in patients with diabetes [25]. Some studies suggest that sulfonylureas increase the risk of stroke, as tolbutamide has been shown to exacerbate neuronal damage in ischemia models, including a mouse middle cerebral artery occlusion (MCAO) model and an *in vitro* neuronal oxygen–glucose deprivation (OGD) model [26].

However, our findings align with those of a previous study that employed a two-sample MR approach, colocalization analyses, and independent blood glucose GWAS summary data to explore the associations between antidiabetic medications and stroke risk. This study demonstrated that sulfonylureas were linked to a decreased risk of both stroke and ischemic stroke, which is consistent with our findings, whereby elevated *KCNJ11* (the target gene of sulfonylureas) expression is associated with an increased risk of AIS [27]. Although the *KCNJ11* colocalization analysis in our study (PPH4 <0.6) did not reach statistical significance, the persistent MR signal supports a causal link, warranting a mechanistic investigation.

In addition to reducing the incidence of stroke, several studies have indicated that sulfonylureas may offer potential benefits for patients with diabetes experiencing acute ischemic stroke. These benefits include associations with reduced stroke severity and decreased mortality rates [28, 29]. Clinical trial results have demonstrated that sulfonylurea compounds, particularly glibenclamide, have a significant effect on reducing cerebral edema in patients with stroke. A randomized, double-anonymized, placebo-controlled GAMES-RP phase 2 trial revealed that intravenous glibenclamide significantly reduced biomarkers of cerebral edema and showed a non-significant mortality trend. However, intravenous glibenclamide failed to meet the primary endpoint established by the trial in patients with large hemispheric infarction. Nonetheless, compared with the placebo, glibenclamide significantly improved survival and tended to improve functional outcomes at 90 days and 12 months among patients aged ≤70 years with large hemispheric infarction [30, 31]. A retrospective cohort study revealed that pre-admission sulfonylurea use in diabetic patients with acute spontaneous intracerebral hemorrhage (ICH) was independently associated with smaller admission hematoma volumes and improved functional outcomes at discharge [32]. Another study demonstrated that intravenous glyburide is related to attenuation of vasogenic edema biomarkers (reduced T2 fluid attenuated inversion recovery (T2 FLAIR) signal intensity, diminished apparent diffusion coefficient (ADC) pseudonormalization, and lower plasma matrix metalloproteinase (MMP-9) levels) in patients with large hemispheric stroke [33]. Additionally, two studies demonstrated that the sulfonylurea compound glibenclamide could mitigate damage induced by ischemia‒reperfusion (IR) injury in the hippocampus [34] and reduce cerebral edema and the infarct volume in rats subjected to MCAO [35]. Consequently, the impact of sulfonylureas on stroke incidence and poststroke recovery, including functional outcomes and mortality, warrants further investigation.

### 4.2 SLC5A2 and SGLT2 Inhibitors

SGLT2, which is encoded by the *SLC5A2* gene, is highly expressed in the proximal renal tubules, facilitating the active transport of D-glucose against its concentration gradient across the electrochemical gradient of sodium ions. SGLT2 inhibitors, which inhibit glucose reabsorption, are oral antidiabetic drugs that successfully reduce serum glucose levels. Our study revealed that a greater risk of ischemic stroke was linked to higher *SLC5A2* gene expression levels. This result is consistent with those of earlier studies showing that SGLT2 inhibitors could lower the incidence of stroke [36, 37, 38, 39, 40].

The EMPA-REG OUTCOME trial reported that empagliflozin, a SGLT2 inhibitor, significantly reduced the risk of cardiovascular mortality, all-cause mortality, and heart failure hospitalization in high cardiovascular risk patients with T2D compared to placebo [36]. A secondary analysis of the EMPA-REG OUTCOME trial demonstrated that empagliflozin consistently reduced cardiovascular risk in patients with T2D and atherosclerotic cardiovascular disease [37]. Lv *et al*. [38] investigated the causal relationship between SGLT1/2 inhibition and clinical signs of cerebral small vascular disease (CSVD) via a two-sample, two-step MR approach. By altering 4-acetamidobutanoate levels and cholesterol metabolism, SGLT2 inhibitors reduced the risk of SVS and increased the integrity of the white matter architecture [38]. To evaluate the association between SGLT2 inhibition and cardiovascular disease (CVD), as well as the mediating effects of blood lipids, Li *et al*. [39] performed a two-sample, two-step MR investigation. SGLT2 inhibition via non-high-density lipoprotein cholesterol (non-HDL-C) was linked to a lower risk of both ischemic stroke and any stroke [39]. A separate meta-analysis revealed that, compared with patients with T2D receiving alternative therapeutic drugs, patients treated with SGLT2 inhibitors had lower risks of stroke, cardiovascular events, and mortality [40]. Moreover, numerous studies have suggested that SGLT2 inhibitors may confer a neuroprotective effect against critical pathological alterations associated with CSVD [41]. Numerous studies examining the underlying mechanisms have documented the effects of SGLT2 inhibitors on blood lipid profiles [42, 43], showing reductions in total cholesterol (TCH), low-density lipoprotein cholesterol (LDL-C), and triglyceride levels [44], which are associated with a greater risk of CVD [45]. In diabetic murine models, SGLT2 inhibitors attenuate atherogenesis, a key contributor to stroke [46]. Moreover, the concurrent use of SGLT1 and SGLT2 inhibitors may significantly reduce the risk of stroke [47].

In addition to decreasing the incidence of stroke, several studies suggest that SGLT2 inhibitors may confer potential benefits in the context of ischemic stroke. In both nondiabetic and diabetic murine models, administering the SGLT2 inhibitor empagliflozin or luseogliflozin was associated with improved neurological outcomes and reduced infarct volumes. These effects may be attributed to the antioxidant, anti-inflammatory, and antiapoptotic properties, as well as a reduction in pericyte loss [48, 49, 50]. Specifically, empagliflozin was shown to mitigate the ultrastructural remodeling of the neurovascular unit and neuroglia induced by T2D, including the attenuation or loss of endothelial tight and adherens junctions within the blood‒brain barrier [51]. Additionally, canagliflozin, another SGLT2 inhibitor, was demonstrated to effectively reduce astrocyte and cerebral swelling in cerebral ischemia [52]. The energy-dependent transport of D-glucose via SGLT2 may exacerbate the energy deficit observed during cerebral ischemia, suggesting that SGLT2 inhibition could provide therapeutic benefits in this context [53].

However, discrepancies also exist among various study findings. Another meta-analysis indicated that SGLT2 inhibitors did not significantly reduce the incidence of ischemic stroke compared with placebo or other oral hypoglycemic agents [54]. A separate meta-analysis suggested a neutral effect of SGLT2 inhibitors on the risk of stroke and associated stroke subtypes while highlighting a potential protective effect against hemorrhagic stroke [55]. These results are consistent with those of other studies [56, 57, 58]. This apparent contradiction may arise from fundamental differences, as our MR analysis serves as a proxy for lifelong SGLT2 inhibition through *SLC5A2* expression, whereas clinical trials evaluate the effects of shorter-term interventions. Therefore, further studies are required to elucidate these findings and clarify the therapeutic potential of SGLT2 inhibitors in stroke prevention and treatment.

### 4.3 INSR

The upregulation of *INSR* expression in the tibial artery was associated with a reduced risk of LAS. Insulin resistance, which is prevalent among patients with ischemic stroke, is linked to increased mortality, increased stroke recurrence rates, and adverse outcomes [59, 60]. Studies have shown that insulin resistance independently predicts atherosclerotic plaque progression. Key underlying mechanisms involve the disruption of insulin signaling across multiple cell types. In endothelial cells, impaired signaling reduces the production of nitric oxide (NO)—a vital vasodilator—and increases the expression of adhesion molecules (e.g., vascular cell adhesion molecule 1 (VCAM-1)), fostering immune cell recruitment and inflammation. Insulin resistance in vascular smooth muscle cells promotes proliferation and migration, contributing to arterial wall thickening. Meanwhile, resistance in macrophages impairs survival and anti-inflammatory functions, hindering cholesterol clearance and increasing plaque inflammation. Furthermore, insulin resistance adversely affects lipid metabolism, elevating triglyceride levels and lowering HDL cholesterol levels, thereby further compounding the risk of atherosclerosis [61]. The insulin-sensitizing agent pioglitazone exerts anti-atherogenic effects by ameliorating insulin resistance and influencing multiple components of insulin resistance syndrome (INRS) [62]. Our findings support the potential benefits of targeting the insulin receptor or using insulin sensitizers in this patient population.

### 4.4 Strengths and Limitations

The strengths and innovations of our study are as follows. First, we employed an SMR analysis, which helps avoid confounding bias and reverse causality, to explore potential drug targets and identify drugs to be repurposed for stroke treatment. Second, repurposing antidiabetic drugs for stroke treatment offers notable advantages, as these drugs have well-established mechanisms of action and clinical data to facilitate faster development of effective therapies. Third, to minimize spurious associations caused by population stratification, we restricted our analyses to individuals of European ancestry, which ensured that differences among populations did not confound the genetic associations observed. However, our study has several limitations. First, this study inferred drug mechanisms solely from gene expression associations, without conducting functional validation, thereby lacking direct mechanistic evidence. Second, this study was limited to evaluating the genetic influences on the incidence of stroke. The effects of antidiabetic medications on poststroke recovery, including functional outcomes and mortality, necessitate further exploration through longitudinal clinical studies. Third, the causal conclusions drawn from MR are contingent upon the following key assumptions: the genetic variants used as instruments must be associated with the drug target, exert an influence on stroke risk exclusively through that target, and not be linked to any confounders in the relationship between the drug and stroke. Additionally, genetic variations reflect the long-term effects of antidiabetic drug targets, which may differ from the effects observed during short-term use of these drugs. Furthermore, the study sample consisted predominantly of individuals of European ancestry; this demographic limitation not only risks introducing bias into the MR results but also restricts the generalizability of our findings to other ethnic groups. Finally, while FI served as a positive control, the absence of FI-associated loci (**Supplementary Table 7**) likely reflects insufficient statistical power in the source GWAS—notably smaller cohort sizes relative to polygenic complexity—rather than biological irrelevance. Future investigations would benefit from large-scale FI-linked GWAS consortia to resolve such modest effects. These limitations should be carefully considered when interpreting our findings.

## 5. Conclusion

Our findings suggest that elevated *SLC5A2* and *KCNJ11* expression levels are associated with an increased risk of ischemic stroke. In contrast, increased *INSR* expression is linked to a reduced risk of LAS. Therapeutic interventions with drugs such as sulfonylureas, SGLT2 inhibitors, and INSR agonists may offer potential benefits for diabetic patients at risk of stroke. This study provides insights into medication strategies for individuals at high risk of stroke who require hypoglycemic agents and supports further investigation into the repurposing of antidiabetic drugs for stroke prevention. Future research should validate these findings and explore the underlying mechanisms involved.

## Data Availability

GWAS summary statistics are publicly available in **Supplementary Table 2**, but individual-level data cannot be deposited publicly due to participant consent restrictions.

## Abbreviations

SMR, summary-based Mendelian randomization; TSMR, two-sample Mendelian randomization; AIS, any ischemic stroke; LAS, large-artery atherosclerotic stroke; T2D, type 2 diabetes; eQTLs, expression quantitative trait loci; HbA1c, glycated hemoglobin; FI, fasting insulin; FG, fasting glucose; 2hGlu, 2-h glucose postchallenge; IVW, inverse-variance weighted; CES, cardioembolic stroke; SVS, small-vessel stroke; IVs, instrumental variables; HEIDI, heterogeneity in dependent instruments; WM, weighted median; K-ATP, ATP-sensitive potassium; MCAO, middle cerebral artery occlusion; OGD, oxygen‒glucose deprivation; IR, ischemia‒reperfusion; CSVD, cerebral small vascular disease; CVD, cardiovascular disease; non-HDL-C, non-high-density lipoprotein cholesterol; TCH, total cholesterol; LDL-C, low-density lipoprotein cholesterol.

## Supplementary Material

Supplementary material associated with this article can be found, in the online version, at https://doi.org/10.31083/RCM40051.
